# Bewältigungsstrategien von Videoconference Fatigue

**DOI:** 10.1365/s40702-023-00963-3

**Published:** 2023-03-30

**Authors:** Victoria Bauer, René Riedl

**Affiliations:** 1 Fakultät für Wirtschaft & Management, Digital Business Institut, Fachhochschule Oberösterreich, Wehrgrabengasse 1–3, 4400 Steyr, Österreich; 2 Institut für Wirtschaftsinformatik – Information Engineering, Johannes Kepler Universität Linz, Altenberger Straße 69, 4040 Linz, Österreich; 3 FWF Der Wissenschaftsfonds, Haus der Forschung, Sensengasse 1, 1090 Wien, Österreich

**Keywords:** Videokonferenzen, Virtuelle Meetings, Videoconference Fatigue, Literaturanalyse, Erschöpfung, Ermüdung, Technostress, Digitaler Stress, Zoom Fatigue, Videoconferencing, Virtual Meetings, Videoconference Fatigue, Literature Review, Exhaustion, Fatigue, Technostress, Digital Stress, Zoom Fatigue

## Abstract

Die COVID-19 Pandemie veränderte global das Berufs- und Privatleben der Menschen und führte zu einer zunehmenden Nutzung von Digitaltechnologien, insbesondere von Videokonferenzsystemen (z. B. Zoom, Webex, Teams, Skype). Trotz der möglichen Vorteile dieser Systeme (z. B. Einsparung von Emissionen durch Reiseverzicht) wird in Wissenschaft und Praxis zunehmend über ein negatives Phänomen berichtet: Videoconference Fatigue (VCF) (synonym oft als Zoom Fatigue bezeichnet). Damit wird die Erschöpfung und Ermüdung beschrieben, die aus der Nutzung von Videokonferenzsystemen resultiert. Im vorliegenden Beitrag berichten wir über eine Analyse der Fachliteratur, deren Ziel es ist, den Stand der Forschung zu Bewältigungsstrategien zu dokumentieren. Die Analyse der Bewältigungsstrategien hilft, das Phänomen VCF besser zu verstehen sowie die Ermüdung und Erschöpfung zu verhindern bzw. zu reduzieren. Weiter sind die hier berichteten Ergebnisse eine Grundlage für zukünftige Arbeiten. Konkret haben wir mit Stand Mai 2022 48 wissenschaftliche Artikel zu VCF identifiziert, von denen sich 37 auch mit Bewältigungsstrategien befassen. Wir gliedern diese Strategien in organisatorische (z. B. Pausen während und zwischen Meetings), persönliche (z. B. Vermeidung von Multitasking) und technologische (z. B. Nutzung des „together mode“ in Teams) Maßnahmen. Ein wesentlicher Befund unserer Analyse ist zudem, dass die Wirksamkeit der Bewältigungsstrategien – mit einer Ausnahme – bislang nicht direkt empirisch untersucht und somit auch nicht nachgewiesen wurde. Dies eröffnet großes Forschungspotenzial für die Zukunft.

**Zusatzmaterial online:**

Zusätzliche Informationen sind in der Online-Version dieses Artikels (10.1365/s40702-023-00963-3) enthalten.

## Abstract (en)

The COVID-19 pandemic has changed people’s professional and private lives globally and has led to an increasing use of digital technologies, especially video conferencing systems (e.g. Zoom, Webex, Teams, Skype). Despite the possible advantages of these systems (e.g. savings in emissions by avoiding travelling), a negative phenomenon has been reported in science and practice: Videoconference Fatigue (VCF) (often synonymously referred to as Zoom Fatigue). This term describes the exhaustion and fatigue that results from the use of videoconferencing systems. In this article we report on an analysis of the academic literature, the aim of which is to document the current state of research on coping strategies. The analysis of coping strategies helps to better understand the phenomenon of VCF and to prevent or reduce fatigue and exhaustion. Furthermore, the results reported here are a basis for future work. Specifically, as of May 2022, we have identified 48 scientific articles on VCF, 37 of which also deal with coping strategies. We divide these strategies into organizational (e.g. taking breaks during and between meetings), personal (e.g. avoiding multitasking) and technological (e.g. use of the “together mode” in Teams). An important finding of our analysis is that with the exception of one paper, the effectiveness of the coping strategies has not yet been empirically examined and thus proven. This opens up enormous future research potential.

## Einleitung

Die im März 2020 von der Weltgesundheitsorganisation (WHO) ausgerufene COVID-19 Pandemie hatte – und hat teilweise noch immer – einen erheblichen Einfluss auf Milliarden von Menschen weltweit. Die Maßnahmen zur Eindämmung der Corona-Pandemie führten insbesondere im Berufsalltag, im Bildungsbereich sowie auch im Privatleben zu Veränderungen in der Kommunikation (Bullock et al. 2021). Persönliche Interaktionen waren nicht mehr im gleichen Umfang wie vor der Pandemie möglich und IT-basierte Kommunikation wurde vermehrt eingesetzt. Besonders Videokonferenzen erfreuten sich zunehmender Beliebtheit, unter anderem deshalb, weil sie der Face-to-Face-Kommunikation ähnlich sind (Fauville et al. 2021; Riedl 2022). Die Nutzung von Videokonferenzen hat sich in allen Branchen verbreitet. Im Bildungsbereich wurden beispielsweise Präsenzveranstaltungen durch Online-Lehre ersetzt und auch in Unternehmen stieg die Zahl der virtuellen Meetings. Auch Arzt-Patienten-Gespräche haben sich zumindest teilweise in den Online-Bereich verlagert. Videokonferenzsysteme wie Zoom, Cisco Webex, Microsoft Teams, Skype sowie Google Hangouts fungierten dabei als Kommunikationsmittel. Seit Beginn der Pandemie stieg die Nachfrage nach diesen Systemen enorm. Die Anzahl der Website-Besuche von zoom.us belief sich beispielsweise im Oktober 2021 auf rund 1,953 Mrd.. Zwei Jahre davor lag die Anzahl der Visits bei 87,4 Mio. (SimilarWeb 2023). Weiter wird auf statista.com prognostiziert, dass sich das weltweite Videokonferenz-Marktvolumen im Jahr 2027 auf rund 11 Mrd. US-Dollar belaufen wird, mehr als doppelt so viel wie 2019 vor Ausbruch der Corona-Pandemie (Statista 2022).

Aus diesen Ausführungen folgt, dass sich die Kommunikation und somit die Interaktion von vielen Menschen weltweit in den letzten Jahren signifikant verändert hat. Es ist daher wichtig, die Wirkungen des vermehrten Einsatzes von Videokonferenzsystemen wissenschaftlich zu untersuchen, um insbesondere auch mögliche negative Wirkungen beschreiben und erklären zu können. Auf diesen Erkenntnissen aufbauend können Interventionen entwickelt werden, die wirksam zur Eindämmung der negativen Folgen beitragen.

Die möglichen Vorteile von Videokonferenzsystemen sind unbestritten (z. B. Ähnlichkeit zu Face-to-Face-Kommunikation bei gleichzeitiger Ortsunabhängigkeit, Einsparung von Emissionen durch Reiseverzicht, reduzierte Reisekosten). Die umfassende Nutzung von Videokonferenzsystemen und der drastische Übergang von physischen zu digitalen Interaktionen hat jedoch ein neues Phänomen hervorgerufen, das als Videoconference Fatigue (VCF) bezeichnet wird (der Begriff „Zoom Fatigue“ wird in der Fachliteratur synonym verwendet). Im Allgemeinen versteht man darunter die stressbedingte Erschöpfung, die sich durch die Nutzung von Videokonferenzsystemen ergibt (Nesher Shoshan und Wehrt 2021). Riedl (2022, S. 157) hat kürzlich auf der Basis einer inhaltsanalytischen Auswertung von zwölf Definitionen eine die inhärenten Phänomeneigenschaften integrierende Begriffsbeschreibung vorgelegt (wörtliche Übersetzung aus dem Englischen): *„Videoconference Fatigue ist definiert als somatische und kognitive Erschöpfung, verursacht durch die intensive und/oder unsachgemäße Verwendung von Videokonferenz-Tools, häufig begleitet von Symptomen wie Müdigkeit, Sorge, Angst, Burnout, Unbehagen und Stress sowie anderen körperlichen Symptomen wie Kopfschmerzen.“* Für eine Darstellung möglicher neurokognitiver Ursachen von VCF verweisen wir auf Arbeiten von Bailenson (2021) und Riedl (2022).

Aufgrund der Tatsache, dass viele Organisationen und Menschen Videokonferenzen mittlerweile massiv in ihr Arbeits- und Privatleben integriert haben und auch Post-Corona ein hoher Verwendungsgrad prognostiziert wird (z. B. aufgrund von Homeoffice; Bolkart 2022), ist die Auseinandersetzung mit diesem neuen Phänomen von hoher Relevanz (Fauville et al. 2021). Weiter wird die Bedeutung der Thematik aus Unternehmenssicht durch Befunde untermauert, die zeigen, dass Videoconferencing sowie die daraus resultierende Ermüdung mit verminderter Arbeitsleistung (Nesher Shoshan und Wehrt 2021), reduzierter Kreativität (Brucks und Levav 2022) und erhöhten Depressions- und Burnout-Tendenzen (Montag et al. 2022) einhergehen können. Nicht zuletzt aufgrund solcher Befunde werden in der Praxis Bewältigungsstrategien gegen VCF dringend benötigt, um die negativen Wirkungen zu vermeiden, zumindest aber abzuschwächen.

In Anbetracht der beschriebenen Ausgangssituation ist es das Ziel des vorliegenden Beitrags, aufzuzeigen, welche VCF-Bewältigungsstrategien bislang in der Fachliteratur beschrieben wurden. Die konkrete Forschungsfrage lautet: *Welche Bewältigungsstrategien gegen Videoconference Fatigue werden in der Fachliteratur beschrieben und mit welcher Häufigkeit? *Bezugnehmend auf Fischer und Riedl (2017, S. 380) ist eine Bewältigungsstrategie ein Denk- oder Verhaltensmuster, das Menschen bzw. Organisationen anwenden, um mit schwierigen Situationen umzugehen und um Belastungen sowie Stress zu vermeiden oder reduzieren.

## Methode der Literaturanalyse

Es wurde eine systematische Literaturanalyse durchgeführt, so wie sie von Webster und Watson (2002) und vom Brocke et al. (2009) vorgeschlagen wird. Um Fachliteratur zu VCF zu identifizieren, erfolgte eine Recherche in folgenden Wissenschaftsdatenbanken: Web of Science, Scopus, IEEE Xplore, ACM Digital Library, AIS eLibrary und EBSCO Host. Für die Recherche wurden folgende Schlüsselwörter in Anlehnung an Riedl (2022) verwendet (Spezifikation Title, Abstract, Keywords): „videoconferenc* fatigue“, „zoom fatigue“, „videoconferenc* stress“, „videoconferenc* exhaustion“ und „virtual meeting fatigue“. Neben den oben genannten Datenbanken wurde zudem eine Suche auf der Grundlage der Software „Publish or Perish“ von Harzing (Version 8.2.3944 Windows GUI Edition) durchgeführt (Angabe Titelwort, Angabe der Datenbank: Google Scholar, letzte Abfrage: 2. Mai 2022). Es wurden 334 Beiträge identifiziert. Daraufhin wurden 103 Duplikate entfernt. Im Anschluss wurden die 231 Beiträge auf die Relevanz für diese Arbeit geprüft. Konkret wurden nach Studium von Titel und Abstract jene 32 Quellen ausgeschlossen, die überhaupt keinen Bezug zu VCF hatten. Im nächsten Schritt wurden die übrigen Artikel im Detail analysiert und Einschluss- und Ausschlusskriterien angewandt. Einschlusskriterium: Der Artikel konzentriert sich auf die Analyse von VCF und/oder untersucht Faktoren, die mit der Erschöpfung von Videokonferenzen zusammenhängen. Ausschlusskriterium: Artikel, die sich mit spezifischen Themen wie der Erschöpfung durch die COVID-19-Pandemie, der Nutzung sozialer Medien, der allgemeinen Mediennutzung und dem Einsatz von Videokonferenzen ohne direkten Forschungsbezug zu VFC befassen. Zudem wurden nicht wissenschaftliche Arbeiten ausgeschlossen (es handelte sich hierbei insbesondere um journalistische Beiträge zu VCF im Internet). So wurden 42 für diese Arbeit relevante Quellen ermittelt.

**Abb. 1:**
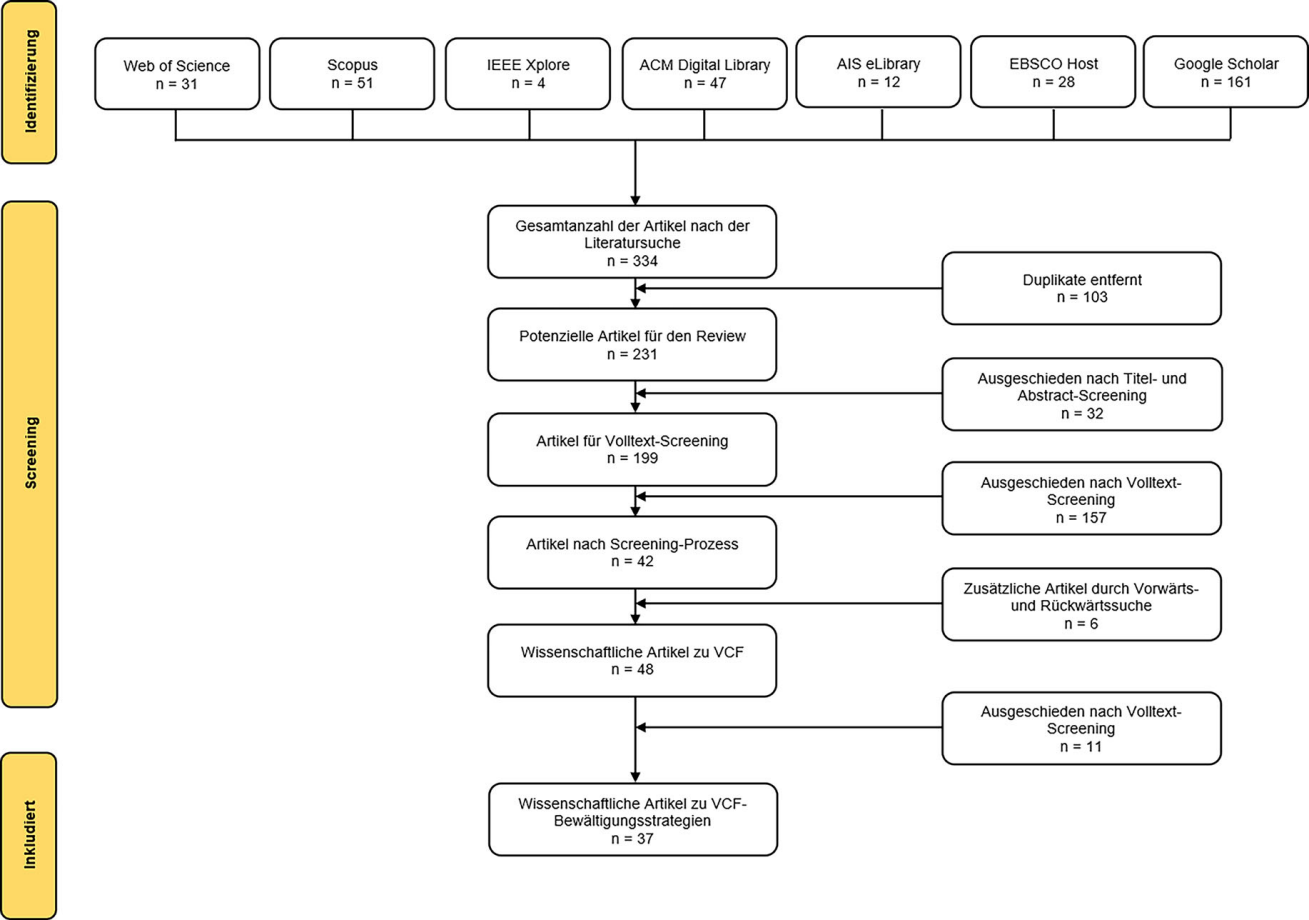
Literaturauswahlprozess. (Adaptierte Version des Prisma 2020 Flow Chart)

Ergänzend dazu wurde eine Rückwärts- und Vorwärtssuche durchgeführt, wie sie von Webster und Watson (2002) empfohlen wird. Durch diesen Schritt konnten noch sechs weitere Quellen identifiziert werden. Die ausgewählten Artikel wurden einer inhaltlichen Volltextanalyse unterzogen. Von den 48 untersuchten Artikeln beschäftigten sich 37 mit VCF-Bewältigungsstrategien. Somit blieben für die im gegenständlichen Beitrag vorgestellten Ergebnisse 37 wissenschaftliche Artikel als Datengrundlage (siehe Anhang). Abb. 1. visualisiert den Literaturauswahlprozess. Zum Rechercheprozess merken die Autoren an, dass explizit auf eine Einschränkung der Recherche auf VCF-Beiträge mit Fokus auf Bewältigungsstrategien in den frühen Suchphasen verzichtet wurde. Dadurch wurde vermieden, dass Beiträge, die zwar Bewältigungsstrategien behandeln, diesen Umstand jedoch nicht in Titel, Abstract oder Keywords zum Ausdruck bringen, nicht identifiziert werden. Im nächsten Schritt wurden die Forschungsergebnisse und die Kernaussagen der Artikel im Hinblick auf Bewältigungsstrategien analysiert.

## Ergebnisse

Nach Sichtung des Datenmaterials entschlossen sich die Autoren, das Kategoriensystem aus Riedl (2022, S. 167) als Grundlage für die Analyse heranzuziehen.^1^ Dabei werden die Maßnahmen in organisatorische, persönliche und technologische Bewältigungsstrategien kategorisiert. Organisatorische Maßnahmen beziehen sich dabei auf Faktoren, die in einer organisatorischen Leitlinie festgelegt werden können. Persönliche Bewältigungsstrategien sind Maßnahmen, die auf der individuellen Ebene vom Benutzer umgesetzt werden können. Die technologischen Maßnahmen beziehen sich auf technische Aspekte und Features der Videokonferenzsysteme. Für die Kategorisierung wurde eine österreichische HR-Managerin eines globalen Technologieunternehmens befragt; diese ordnete die in den folgenden Abschnitten genannten Maßnahmen auf jeweils eine der drei Kategorien zu. Die beiden Autoren des vorliegenden Beitrags sichteten danach die Kategorisierungen und stimmten in allen Fällen mit der Klassifikation überein.

**Abb. 2:**
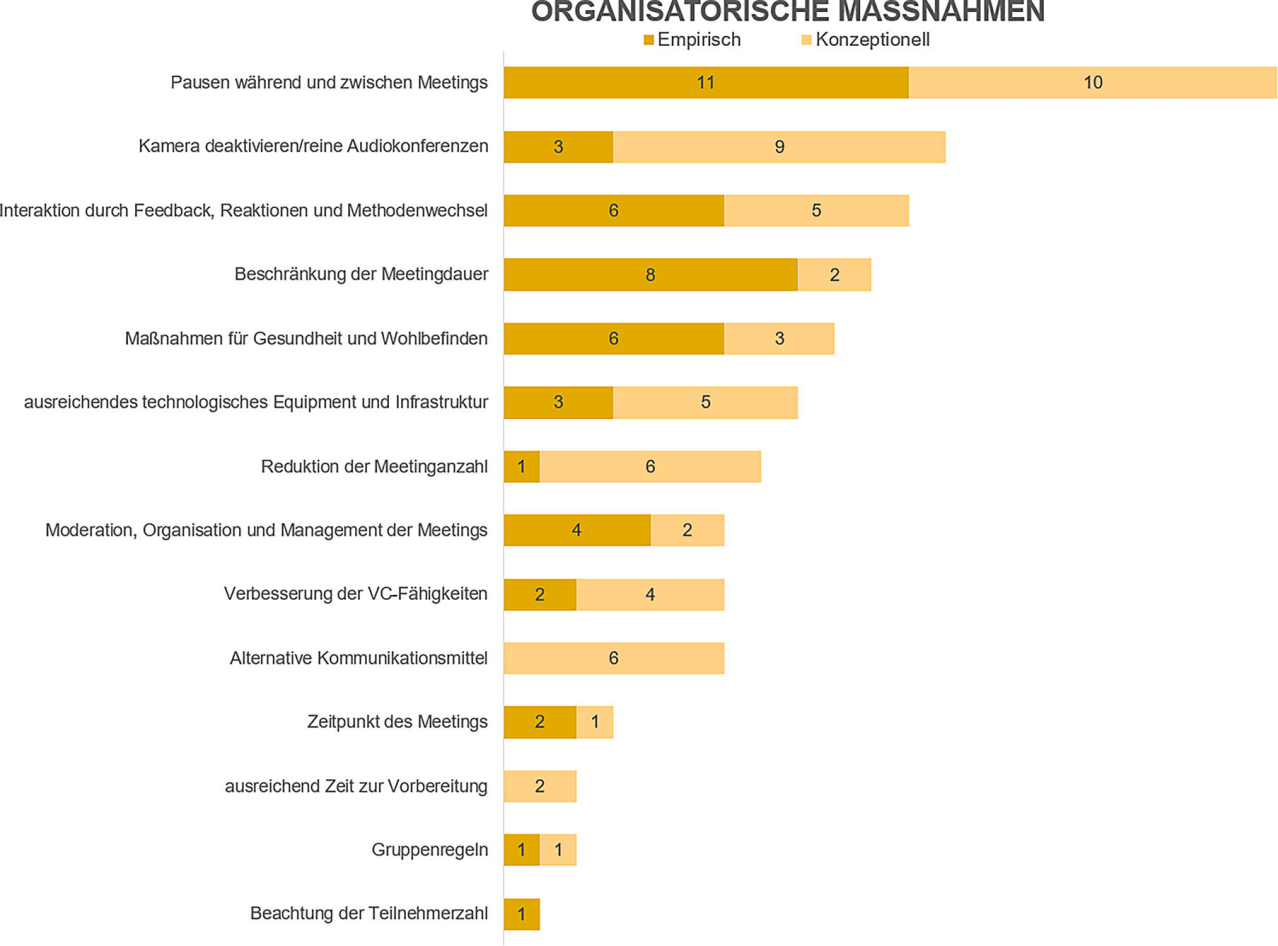
Organisatorische Maßnahmen gegen Videoconference Fatigue

In den folgenden Ausführungen erfolgt auch eine Gruppierung der Bewältigungsstrategien in empirische Studien und konzeptionelle Artikel (in den Abb. 2, 3 und 4 als „empirisch“ und „konzeptionell“ bezeichnet). Ein wesentlicher Befund unserer Analyse ist, dass in vielen Artikeln die (Wirksamkeit der) Bewältigungsstrategien nicht direkt empirisch untersucht wurde(n), sondern in den konzeptionellen Beiträgen die Strategien einfach beschrieben werden und/oder deren Wirksamkeit zwar behauptet, jedoch nicht durch Anwendung anerkannter empirischer Forschungsmethoden nachgewiesen wird.

**Abb. 3:**
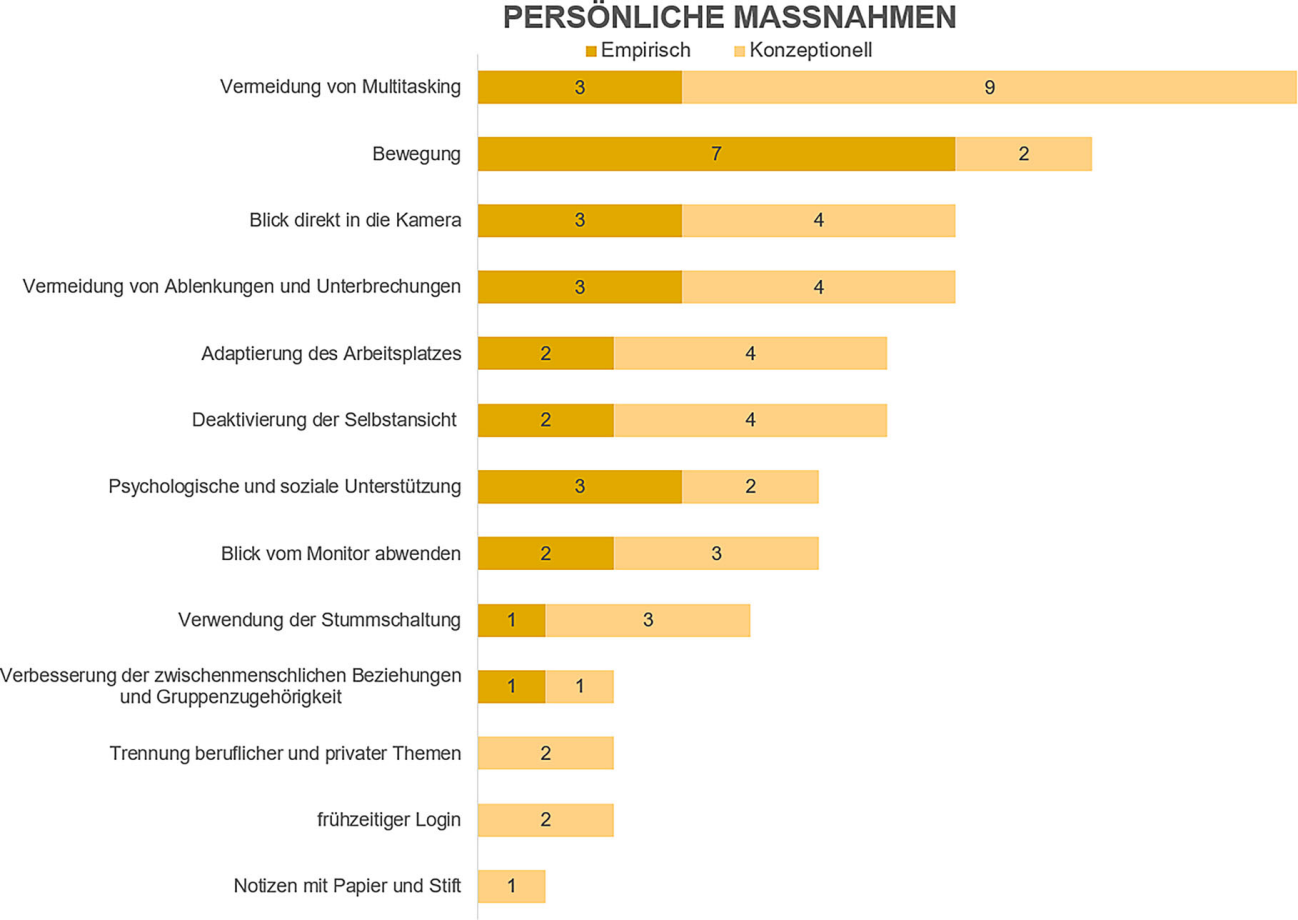
Persönliche Maßnahmen gegen Videoconference Fatigue

**Abb. 4:**
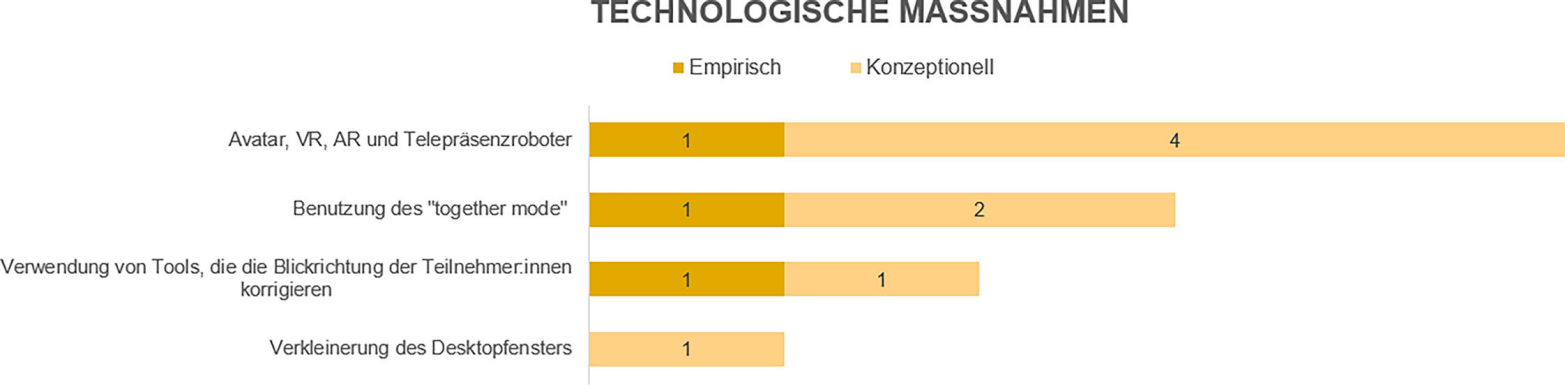
Technologische Maßnahmen gegen Videoconference Fatigue

### Organisatorische Maßnahmen

Insgesamt wurden 14 Faktoren den organisatorischen Maßnahmen zugeordnet. Abb. 2 zeigt einen Überblick über diese Bewältigungsstrategien. Die entsprechende Literaturmatrix ist im Onlinematerial dargestellt.

#### Pausen während und zwischen Meetings

Die am häufigsten genannte Bewältigungsstrategie unter den organisatorischen Maßnahmen ist das Einlegen von Pausen während und zwischen den Videokonferenz-Meetings. Der aktuelle Stand der Forschung zeigt, dass insbesondere die Anzahl und Häufigkeit von Videokonferenzen zu VCF führt. Wenn genügend Pausen eingeplant werden, kann die dadurch entstandene Müdigkeit reduziert werden (Döring et al. 2022). Nach der „Attention Restoration Theory“ sind Pausen hilfreich, um die Erschöpfung und Ermüdung zu verringern. Wenn diese nicht von der jeweiligen Meeting-Organisation vorgesehen sind, sollten die Teilnehmer selbst darauf achten (Bennett et al. 2021). Im Zusammenhang mit Online-Lehre wurde empfohlen, mehrere Pausen einzulegen, um der Erschöpfung und Ermüdung entgegenzuwirken (Asgari et al. 2021). Für Lehrende als auch für Lernende ist es hilfreich, gleich zu Beginn konkrete Pausenzeiten festzulegen und diese dann auch einzuhalten (Bennett et al. 2021).

#### Kamera deaktivieren/reine Audiokonferenzen

Neben Pausen wurden auch das Ausschalten der Kamera und die Durchführung von reinen Audiokonferenzen als organisatorische Maßnahmen gegen VCF genannt. Um Fatigue zu verringern, sollten Ablenkungen und Überreizung so weit wie möglich vermieden werden. Um dies zu erreichen, kann während einer Besprechung vereinbart werden, dass nur die Personen, die gerade sprechen, das Video eingeschaltet haben (Wiederhold 2020). Außerdem sollte den Teilnehmern die Möglichkeit gegeben werden, die Kamera zumindest für eine gewisse Zeit zu deaktivieren (Brown 2022). Zudem schlägt Bailenson (2021) vor, standardmäßig Audiokonferenzen zu verwenden, um die kognitive Belastung durch die Produktion und Interpretation von nonverbalen Hinweisen zu verringern.

Shockley et al. (2021) zeigen in ihrer Studie, dass die Teilnehmer davon profitieren, wenn sie die Möglichkeit haben, die Kamera auszuschalten. Dabei ist zu beachten, dass dies nicht bedeutet, dass Besprechungen ohne den Einsatz von Kameras automatisch ansprechender sind (Shockley et al. 2021). Das Einschalten der Kamera kann ebenso dazu beitragen, eine virtuelle Nähe zwischen den Teilnehmern zu schaffen und so das Gefühl der Zugehörigkeit zu stärken. Obwohl durch die Kamera ein gewisser Druck besteht, aufmerksam zu sein und man sich mehr Gedanken über das Aussehen, die Umgebung und die Mimik macht, können dadurch auch Vorteile entstehen (Bennett et al. 2021).

#### Interaktion durch Feedback, Reaktionen und Methodenwechsel

Als weitere Maßnahme gegen VCF wurden die Einbeziehung der Teilnehmer und die Interaktion in Besprechungen erwähnt. Dies kann unter anderem durch Umfragen, möglichst viele abwechslungsreiche Methoden und Fragen an das Publikum geschehen (Bennett et al. 2021). Die Mitwirkung der Teilnehmer kann durch die Aufforderung zur Verwendung von Reaktionen, direktes Feedback und Kommentare ermöglicht werden (Massner 2021). Darüber hinaus kann das Interesse der Teilnehmer durch unerwartete Informationen und Inhalte geweckt werden (Rößler et al. 2021). Durch die aktive Teilnahme von Personen in einer virtuellen Besprechung kann verhindert werden, dass Multitasking betrieben wird (also parallel zur Videokonferenz an anderen Aufgaben gearbeitet wird, z. B. auf einem zweiten Bildschirm). Auf diese Weise lässt sich in weiterer Folge die Müdigkeit verringern (Döring et al. 2022).

#### Beschränkung der Meetingdauer

Die Dauer von Besprechungen sollte begrenzt werden. Eine kürzere Dauer von Meetings kann die Ermüdung verringern. Als Richtwert wird vorgeschlagen, dass Besprechungen nicht länger als eine Stunde dauern sollten (Amponsah et al. 2021).

#### Maßnahmen für Gesundheit und Wohlbefinden

In den analysierten Artikeln wird die Einführung von Maßnahmen zur Verbesserung der Gesundheit und des Wohlbefindens als Bewältigungsstrategie für VCF genannt. Unternehmen und Bildungseinrichtungen können Initiativen zur Stärkung der Gesundheit und des Wohlbefindens von Personen anbieten. Dazu gehören Kurse zu Achtsamkeit, Mediation und Bewegung (Bullock et al. 2021). In diesem Zusammenhang können auch Beruhigungstechniken (z. B. bewusste Atmung und Übungen zur Muskelentspannung) eingesetzt werden, um Stress zu reduzieren. Durch Mediation kann die psychische Gesundheit gestärkt und eine gute Schlafqualität für Menschen gewährleistet werden (Salim et al. 2022).

#### Ausreichendes technologisches Equipment und Infrastruktur

Forschungsbefunde zeigen, dass technische Probleme zu Stress und in weiterer Folge zu Erschöpfung führen (für einen Review, siehe Riedl 2013). Um dem vorzubeugen, sollte auf eine angemessene technische Ausstattung und eine gute Infrastruktur geachtet werden. Besonders bedeutsam sind eine zuverlässige und schnelle Internetverbindung und ein passendes Soundsystem (Nesher Shoshan und Wehrt 2021).

Weiter können Weitwinkelkameras sowie hochwertige Mikrofone und Lautsprecher eingesetzt werden, um den Stress durch mögliche Verständnisprobleme zu reduzieren (Döring et al. 2022). Darüber hinaus wird empfohlen, eine externe Kamera und eine externe Tastatur zu verwenden (was im Regelfall bei der Nutzung eines Laptops nicht der Fall ist). Dies ermöglicht eine größere Flexibilität in Bezug auf die Sitzposition und schafft eine gewisse Distanz zwischen den Teilnehmern einer Videokonferenz und dem verwendeten Bildschirm (Bailenson 2021; Bullock et al. 2021).

#### Reduktion der Meetinganzahl

Für die Vermeidung von VCF ist es nützlich, abzuschätzen, in welchen Situationen es tatsächlich notwendig ist, eine Videokonferenz durchzuführen. Die Verringerung der Anzahl von virtuellen Meetings führt zu einer geringeren Ermüdung (Mamtani et al. 2021). Vor allem die Beschränkung der Anzahl der Besprechungen an einem Tag kann gegen VCF wirksam sein (Massner 2021). Die Begründung hierfür liegt darin, dass beim Videoconferencing – im Gegensatz zu Kommunikation von Angesicht zu Angesicht – oftmals mehr kognitive Ressourcen benötigt werden, was insbesondere mit rascherer hirnphysiologischer Erschöpfung einhergehen kann (Riedl 2022). Außerdem sollte nicht jedes Meeting virtuell stattfinden, sofern andere Möglichkeiten des Austauschs bestehen (Brown Epstein 2020).

#### Moderation, Organisation und Management der Meetings

Die Moderation, Organisation und das Management eines Meetings zählen auch zu den Strategien, die eingesetzt werden können, um VCF entgegenzuwirken. In einem Meeting kann eine angemessene Moderation dazu führen, dass die Teilnehmer weniger erschöpft sind. Neben dem gezielten Einsatz von Pausen und einem Zeitmanagement ist ein Moderator dafür verantwortlich, die Tagesordnung einzuhalten und mögliche Diskussionen zu leiten, um nicht von den wesentlichen Themen abzuweichen (Amponsah et al. 2021).

Die richtige Organisation und das Management von Besprechungen sind sowohl für reale als auch für virtuelle Treffen wichtig. Ziel ist es, die Effektivität der Besprechungen zu erhöhen. Dies kann durch gutes Zeitmanagement, Pausenplanung und Moderation erreicht werden (Nesher Shoshan und Wehrt 2021). Neben der Moderation eines Meetings, hat auch die positive Einstellung der vortragenden Person einen Einfluss auf VCF. Grund dafür ist, dass sich die Begeisterung auf die Zuhörer überträgt und zu einem positiven Eindruck führt (Rößler et al. 2021).

#### Verbesserung der VC-Fähigkeiten

In der Fachliteratur wird darüber berichtet, dass die Verbesserung der Fähigkeiten in Bezug auf die Nutzung von Videokonferenzsystemen zur Verringerung von VCF beitragen kann. Die Bereitstellung von entsprechenden Trainings inklusive Dokumentationen zu den Systemen dient der Komplexitätsminderung und kann somit den Stress reduzieren. Schulungen erhöhen zudem das Vertrauen in die Nutzung von Videokonferenzsystemen (Bullock et al. 2021). Darüber hinaus kann die Unterstützung und ein Online-Support für die Nutzer von Videokonferenzsystemen hilfreich sein. Für die Nutzung können zudem Leitlinien entwickelt und angeboten werden (Usta Kara und Ersoy 2022).

#### Alternative Kommunikationsmittel

Eine weitere Möglichkeit zur Reduktion von VCF ist die Nutzung von Kommunikationsmitteln abseits von Videolösungen (Bailenson 2021). Vor allem während der COVID-19 Pandemie wurden Videokonferenzen für viele Menschen zum Standardkommunikationskanal. Für bestimmte Zwecke sind jedoch Telefonate ausreichend; Riedl (2021, S. 215) schreibt in diesem Zusammenhang vom Sprachimperativ, der unter anderem besagt, dass „die Qualität der Übermittlung von Inhalten beim Telefonieren nur unwesentlich schlechter ist als bei der Kommunikation von Angesicht zu Angesicht.“ Primäre Ursache dafür ist die durch die Semantik hervorgerufene enorme Wirksamkeit der menschlichen Sprache. So kann die Zahl der Videokonferenzen reduziert werden und damit auch die Erschöpfung. Neben einem Telefonat ist häufig auch E‑Mail ein zweckmäßiges Kommunikationsmittel (Wicks 2021). Die Wahl des Kommunikationsmittels wird im Regelfall situationsspezifisch entschieden und hängt von mehreren Faktoren ab – Beispiele sind: Komplexität des Sachverhalts, Notwendigkeit oder Erwünschtheit einer schriftlichen Dokumentation zum Sachverhalt, Dringlichkeit des Sachverhalts, Vermeiden einer Unterbrechung beim Kommunikationspartner, räumliche Distanz zwischen den Kommunikationspartnern sowie die Anzahl der am Kommunikationsprozess beteiligten Personen.

#### Zeitpunkt des Meetings

Zur Verringerung der Erschöpfung ist es auch hilfreich, den Zeitpunkt der Besprechungen zu berücksichtigen. Da das Ausmaß an körperlicher Energie von Menschen im Laufe des Tages schwankt, sind Meetings zu bestimmten Zeiten des Tages von Vorteil. Die Ergebnisse einer quantitativen Studie zeigen, dass bestimmte Tageszeiten mit höherer Müdigkeit einhergehen. Videokonferenzen sollten daher zu einem Zeitpunkt abgehalten werden, an dem eine Mehrheit der Teilnehmer am aktivsten ist, was der Studie zufolge zu Beginn des Tages der Fall ist (Bennett et al. 2021). Außerdem sollte bei der Planung von Besprechungen nach Möglichkeit versucht werden, diese über die Woche zu verteilen, anstatt Meetings blockweise durchzuführen (Shklarski et al. 2021).

Die restlichen Bewältigungsstrategien wurden in weniger als drei Artikel genannt, weshalb diese in diesem Beitrag nicht näher ausgeführt werden. Dazu gehören ausreichende Zeit für die Vorbereitung, Gruppenregeln, sowie die Beachtung der Teilnehmerzahl (nicht zu viele; was „zu viele“ bedeutet, ist situationsspezifisch zu beurteilen).

### Persönliche Maßnahmen

Es wurden 13 Faktoren den persönlichen Maßnahmen zugeordnet. Die entsprechende Literaturmatrix ist im Onlinematerial dargestellt. Abb. 3 zeigt einen Überblick über diese Bewältigungsstrategien.

#### Vermeidung von Multitasking

Die am häufigsten genannte Bewältigungsstrategie bei den persönlichen Maßnahmen ist die Vermeidung von Multitasking. Die zusätzliche Aufmerksamkeit, die das Multitasking erfordert, führt zur Erschöpfung. Aus diesem Grund sollten die Teilnehmer versuchen, Aktivitäten, die nichts mit der Besprechung zu tun haben, zu vermeiden, zumindest aber zu reduzieren (Wiederhold 2020). Aus diesem Grund sollten Videokonferenzteilnehmer auf die parallele Benutzung von Mobiltelefonen verzichten (Bullock et al. 2021). Außerdem sollten nicht benötigte Browserfenster geschlossen werden, um nicht in Versuchung zu geraten, sich ablenken zu lassen (Ngien und Hogan 2022).

#### Bewegung

Eine weitere Maßnahme gegen VCF ist die körperliche Bewegung zwischen Besprechungen. Da Videokonferenzen in der Regel im Sitzen abgehalten werden, lässt die Konzentration nach einer Weile nach und man wird zunehmend passiver. Dies kann durch Bewegung unterbrochen werden, um die Aufmerksamkeit zu erhöhen. Bereits nach 30 min Meetingdauer empfiehlt es sich, die Körperposition zu verändern (z. B. aus dem Sitzen aufzustehen), um sich zu dehnen und zu bewegen (Peper et al. 2021). Auch vor dem Hintergrund des Praktizieren solcher Maßnahmen ist das (zumindest temporäre) Ausschalten der eigenen Kamera zweckmäßig.

Neben der Steigerung von Konzentration und Aufmerksamkeit kann physische Aktivität dazu beitragen, körperliche Ermüdung zu verringern. So können neben Dehnübungen auch Aktivitäten wie Laufen und Spazierengehen im Freien zwischen den Videokonferenzen dazu beitragen, VCF zu verringern (Shklarski et al. 2021; Usta Kara und Ersoy 2022). Riedl (2021) fasst Forschungsbefunde aus Medizin und Physiologie zusammen, welche die Wirksamkeit von Bewegung als Maßnahme gegen Technostress belegen.

#### Blick direkt in die Kamera

Eine weitere Möglichkeit, VCF zu vermeiden bzw. zu reduzieren, ist der direkte Blick in die Kamera. Es wird empfohlen, den Blick nicht auf die anderen Teilnehmer zu richten. Grund dafür ist, dass dies zu einer erhöhten Aufmerksamkeit führt, da man versucht, das Geschehen der oft verwendeten Galerieansicht zu verarbeiten. Demnach ist es ratsam, wenn möglich direkt in die Kamera zu blicken. Zum einen führt dies dazu, dass man gegenüber den anderen Personen engagierter wirkt und zum anderen wird die Ermüdung dadurch reduziert (Wicks 2021). Außerdem verhindert der Blick in die Kamera, dass sich die Teilnehmer in Besprechungen selbst beobachten (Usta Kara und Ersoy 2022).

#### Vermeidung von Ablenkungen und Unterbrechungen

Eine weitere Bewältigungsstrategie ist die Vermeidung von Ablenkungen und Unterbrechungen. In einer Videokonferenz ist es möglich, virtuelle Hintergründe zu verwenden. Diese können jedoch ablenkend wirken. Obwohl sie auch einen Unterhaltungsfaktor bieten, sollten sie nicht übermäßig eingesetzt werden (Brown 2022). Die Umgebung sollte ebenso von den Teilnehmern kontrolliert werden, damit keine Unterbrechungen auftreten, was insbesondere im Homeoffice der Fall sein kann (Massner 2021).

#### Adaptierung des Arbeitsplatzes

Es wurde auch die angemessene Einrichtung des Büroarbeitsplatzes als Maßnahme zur Verringerung der Ermüdung genannt. Dazu gehört unter anderem die Ergonomie der Büromöbel, weshalb ein Bürostuhl mit einer geeigneten Rückenlehne verwendet werden sollte (Brown Epstein 2020). Zur Einrichtung des Arbeitsplatzes für eine Videokonferenz wird auch empfohlen, den Bildschirm auf einer passenden Höhe zu platzieren. Außerdem sollten Personen darauf achten, dass sie für andere Teilnehmer gut sichtbar sind und das Gesicht ausreichend beleuchtet wird (Wiederhold 2020).

#### Deaktivierung der Selbstansicht

Studien zeigen, dass die permanente Selbstansicht bei Videokonferenzen zu VCF führt. Um dies zu vermeiden, wird vorgeschlagen, den eigenen Videostream auszublenden. Das Ausschalten der Selbstansicht kann Faktoren wie die Unzufriedenheit mit dem eigenen Aussehen und die sogenannte „Spiegelangst“ („Mirror Anxiety“) verringern (Ratan et al. 2021). Es wird daher in der Fachliteratur vorgeschlagen, dass Videokonferenzsysteme die Selbstansicht automatisch ausblenden sollten, zumindest nach einer gewissen Zeit (Bailenson 2021).

#### Psychologische und soziale Unterstützung

Psychologische und soziale Unterstützung sind ebenfalls hilfreich bei der Bewältigung der Erschöpfung. Die Auswirkungen von VCF auf die Psyche können durch Gespräche mit Freunden und Familie besser verarbeitet werden (Shklarski et al. 2021; Bullock et al. 2021). Zudem helfen psychologische und soziale Unterstützung beim Umgang mit der Erschöpfung grundsätzlich (Usta Kara und Ersoy 2022). Außerdem soll die Angst vor sozialer Interaktion abgebaut werden, um VCF zu reduzieren. Insbesondere sollte vermieden werden, den eigenen Selbstwert über die Meinung anderer zu definieren (Ngien und Hogan 2022).

#### Blick vom Monitor abwenden

Eine weitere Maßnahme zur Verringerung von VCF besteht darin, die ständige Aufmerksamkeit und Konzentration zu unterbrechen. Dazu ist es erforderlich, sich physisch vom Computer zu entfernen sowie den Blick vom Bildschirm abzuwenden (Ebner und Greenberg 2020). Dabei ist es auch möglich, zwischen den Sitzungen die Umgebung zu wechseln, um die anhaltende und intensive Aufmerksamkeit zu unterbrechen (Bullock et al. 2021). Außerdem werden die Augen bei Videokonferenzen durch den Bildschirm stark beansprucht, was zu einer erhöhten Ermüdung führt. Um die Belastung der Augen zu reduzieren und Stress abzubauen, ist es hilfreich, während Besprechungen den Blick vom Bildschirm abzuwenden und immer wieder mal für kurze Zeit in die Ferne zu schauen. Das entspannt die Augenmuskeln und unterbricht den visuellen Stress (Peper et al. 2021).

#### Verwendung der Stummschaltung

Als Bewältigungsstrategie von VCF wird weiter die „Stummschaltung“ beschrieben. Ein Grund für die Erschöpfung bei Videokonferenzen liegt unter anderem in der erhöhten Aufmerksamkeit, die während einer Besprechung erforderlich ist. Hintergrundgeräusche machen es schwierig, die Aufmerksamkeit aufrechtzuerhalten. Die Stummschaltung des Mikrofons kann helfen, Ablenkungen zu reduzieren (Bennett et al. 2021).

Die restlichen Bewältigungsstrategien wurden in weniger als drei Artikel erwähnt, weshalb diese nicht näher ausgeführt werden. Dazu gehören die Verbesserung der zwischenmenschlichen Beziehungen und Gruppenzugehörigkeit (z. B. durch Vertrauensbildungsprozesse vor dem virtuellen Meeting), die Trennung beruflicher und privater Themen, ein frühzeitiger „Login“ sowie Notizen mit Papier und Stift zu machen.

### Technologische Maßnahmen

Vier Faktoren wurden den technologischen Maßnahmen zugeordnet. Abb. 4 zeigt einen Überblick über diese Bewältigungsstrategien. Die entsprechende Literaturmatrix ist im Onlinematerial dargestellt.

#### Avatar, VR, AR und Telepräsenzroboter

Zur Reduzierung der selbstfokussierten Aufmerksamkeit sowie der Unzufriedenheit mit der eigenen Erscheinung (bei Einschaltung des eigenen Videostreams beim Videoconferencing) können Avatare zum Einsatz kommen. Diese werden bereits in Videospielen, aber auch in virtuellen Meetings eingesetzt. Avatare reduzieren die Selbstfokussierung (Wiederhold 2021). Im Gegensatz zur Deaktivierung der Selbstsicht können sich die Teilnehmer aber weiterhin selbst beobachten. Damit entfallen Probleme wie die Übertragung möglicher ungewollter Hintergrundaktivitäten (Ratan et al. 2021). Wiederhold (2020) erwähnt den Einsatz von Virtual Reality (VR) und Augmented Reality (AR), um VCF zu verringern. Der Vorteil virtueller Konferenzräume – so wie sie auch in Metaverse-Anwendungen Verwendung finden – ist ihre Ähnlichkeit zu persönlichen Treffen. Die Verwendung von Tools wie Spatial, die eine räumliche Nähe schaffen, zählt zu den VCF-Bewältigungsstrategien (Wiederhold 2020).

Auch der Einsatz von Telepräsenzrobotern (siehe z. B. Brooks 2002) wird als Strategie gegen VCF beschrieben. Ähnlich wie bei der Verwendung von Avataren können Telepräsenzroboter die selbstfokussierende Aufmerksamkeit verringern. Dabei handelt es sich um einen selbstfahrenden Roboter, der mit einer Webcam und einem Mikrofon ausgestattet ist. Diese Technologie ermöglicht den Teilnehmern einer Videokonferenz, sich mit Hilfe des Roboters in einem physischen Raum zu bewegen und ihre Aufmerksamkeit selbst zu lenken (Wicks 2021).

#### Benutzung des „together mode“

Ein Faktor, der bei der Bewältigung von Erschöpfung durch Videokonferenzen eine Rolle spielt, ist die Verwendung von Software zur Nachahmung persönlicher Interaktion. Microsoft Teams bietet beispielsweise den „together mode“ an, Zoom das Feature „immersive view“. Diese Features schaffen den Eindruck, als säßen alle Teilnehmer in einem Raum. Da durch diese Funktion das typische Erscheinungsbild mit einer Kachel je Person entfällt, wird die Reizüberflutung reduziert und das Zusammengehörigkeitsgefühl kann gesteigert werden (Brown Epstein 2020).

#### Verwendung von Tools, die die Blickrichtung der Teilnehmer korrigieren

Eine weitere Maßnahme gegen VCF ist die Verwendung von Softwarefunktionen, die ein persönliches Gespräch nachahmen. Um den bei Videokonferenzen fehlenden Augenkontakt herzustellen, können Tools eingesetzt werden, die die Blickrichtung von Personen in die Richtung der Kamera korrigieren (Riedl 2021). Beispielsweise hat Microsoft kürzlich ein neues Feature mit der Bezeichnung „Eye Contact“ vorgestellt, das auf Künstlicher Intelligenz beruht und die Blickrichtung bei Videogesprächen so korrigiert, dass ein direkter Blick in die Kamera durch die Gesprächsteilnehmer wahrgenommen wird. Apple bietet bei Videogesprächen via FaceTime ein gleichnamiges Feature an (in früheren iOS-Versionen als „FaceTime Attention Correction“ bezeichnet).

#### Verkleinerung des Desktopfensters

Eine andere Ursache von VCF bezieht sich auf die Interaktion mit vielen Gesichtern (Bailenson 2021). Um diesen negativen Effekt zu verringern, ist es ratsam, das Fenster der Videokonferenz zu verkleinern. Dies erhöht den wahrgenommenen Abstand zu den Gesichtern und wirkt daher stress- und ermüdungsreduzierend (Brown Epstein 2020; Riedl 2022).

## Implikationen für Praxis und Wissenschaft

Aufgrund der Tatsache, dass Videokonferenzen immer mehr auch zu einem festen Bestandteil des Arbeitslebens werden und die daraus resultierenden negativen Folgen insbesondere aus Unternehmenssicht weitreichend sind, können die in diesem Beitrag identifizierten Bewältigungsstrategien in der Praxis nützlich sein, um VCF zu reduzieren bzw. im Idealfall gänzlich zu vermeiden. Im organisatorischen Umfeld können HR-Verantwortliche den Einsatz der vorgestellten Maßnahmen planen und umsetzen; insbesondere können unternehmensweite Richtlinien für den Umgang mit Videokonferenzen entwickelt und erprobt werden. Ebenso können die Benutzer von Videokonferenz-Tools die beschriebenen persönlichen Maßnahmen im Arbeitsalltag umsetzen, um die Ermüdung zu reduzieren. Die technologischen Maßnahmen beschreiben jene Funktionalitäten, die aktuell einen Beitrag zur Reduktion von VCF leisten können. Hersteller von Videokonferenz-Tools sollten mit Nachdruck an der Weiterentwicklung existierender sowie an der Entwicklung neuer Features arbeiten, die einen wirksamen Beitrag zur Reduktion von VCF leisten können. Beispielsweise könnte ein Feature implementiert werden, das nach einer bestimmten Zeit automatisiert eine Nachricht am Bildschirm einblendet, die zu einer Pause auffordert. Damit kann den negativen Folgen von Videoconferencing vorgebeugt werden.

Die Häufigkeit und Dauer von Meetings weisen einen positiven Zusammenhang mit der Ermüdung und Arbeitsbelastung von Mitarbeitern auf, dies gilt auch bei physischen Besprechungen (Luong und Rogelberg 2005). Deshalb wird offensichtlich, dass die hier vorgestellten Bewältigungsstrategien teilweise auch auf physische Meetings angewendet werden können und sollen. Zudem ergibt sich aus den hier vorgestellten Befunden die bislang empirisch unbeantwortete Frage, ob es beispielsweise in Bezug auf Fatigue-Reduktion wirksamer ist, während und zwischen Meetings Pausen zu machen oder die Kamera zwischendurch zu deaktivieren. Zukünftige Arbeiten sind notwendig, um entsprechende Forschungserkenntnisse zu erarbeiten. Diese leisten dann einen Beitrag zur Beantwortung der Frage, welche die wirksamsten Bewältigungsstrategien sind.

## Limitationen und Fazit

Diese Arbeit bietet auf der Basis einer systematischen Literaturanalyse einen Überblick über Strategien, die einen Beitrag zur Vermeidung bzw. Reduktion von VCF leisten können. Konkret beantwortet der vorliegende Beitrag die Frage, welche Bewältigungsstrategien gegen VCF in der Fachliteratur beschrieben werden und mit welcher Häufigkeit dies geschieht. Bei der Interpretation der hier präsentierten Befunde ist zu beachten, dass selbst in Arbeiten, in denen empirische Forschungsmethoden zum Einsatz gekommen sind, die Wirksamkeit von Bewältigungsstrategien nicht direkt untersucht wurde (mit Ausnahme von Bennett et al. 2021). Zukünftige Forschung sollte daher darauf abzielen, diese Lücke zu schließen. Die Ergebnisse dieser Arbeit sollen dabei als Grundlage dienen. Insbesondere stellen die in den Abb. 2, 3 und 4 zusammengefassten organisatorischen, persönlichen und technologischen Bewältigungsstrategien eine Grundlage dar, aus der Forschende Ideen für Untersuchungsobjekte gewinnen können. Beispielsweise ist die am häufigsten genannte Strategie im organisationalen Bereich „Pausen während und zwischen Meetings“. Zukünftige Forschung könnte unter anderem untersuchen, wie lange solche Pausen sein sollten, um durch Videoconferencing angestiegene Ermüdungswerte (z. B. durch Fragebogen oder physiologisch gemessen; Riedl 2022) wieder auf das Ausgangsniveau zu bringen.

## Supplementary Information

Literaturklassifikation nach organisatorischen, persönlichen und technologischen Maßnahmen

## Anhang

**Tab. 1:** Datengrundlage: 37 Artikel

Nr	Quelle	Titel	Publikationsmedium
1	Amponsah et al. (2021)	Academic experiences of “Zoom-Fatigue” as a virtual streaming phenomenon during the COVID-19 pandemic	International Journal of Web-Based Learning and Teaching Technologies
2	Asgari et al. (2021)	An observational study of engineering online education during the COVID-19 pandemic	PLOS ONE
3	Bailenson (2021)	Nonverbal overload: A theoretical argument for the causes of Zoom Fatigue	Technology, Mind, and Behavior
4	Bayindir und Gökce (2022)	The levels of coping with online course (Zoom) Fatigue of preservice teachers in the emergency remote teaching process	ITTES 2021
5	Bennett et al. (2021)	Videoconference Fatigue? Exploring changes in fatigue after videoconference meetings during COVID-19	Journal of Applied Psychology
6	Brown (2022)	Lessons learned from pandemic teaching	SIGUCCS ’22: Proceedings of the 2022 ACM SIGUCCS Annual Conference
7	Brown Epstein (2020)	Virtual Meeting Fatigue	Journal of Hospital Librarianship
8	Bullock et al. (2021)	“All Zoomed Out”: Strategies for addressing zoom fatigue in the age of COVID-19	The Learning Ideas Conference
9	Chawla (2021)	Zoom fatigue saps grant reviewers’ attention	Nature
10	Collins (2020)	Social distancing as a critical test of the micro-sociology of solidarity	American Journal of Cultural Sociology
11	De Oliveira Kubrusly Sobral et al. (2022)	Active methodologies association with online learning fatigue among medical students	BMC Medical Education
12	Döring et al. (2022)	Videoconference Fatigue: A conceptual analysis	International Journal of Environmental Research and Public Health
13	Ebner und Greenberg (2020)	Designing binge-worthy courses: Pandemic pleasures and COVID-19 consequences	American Journal of Cultural Sociology
14	Hall (2020)	From zoom fatigue to belly breaths: Teaching away from the screen	Teaching Theology and Religion
15	Hopf und Berger (2022)	Zoom-Fatigue – Ein Phänomen im Distance-Learning	R&E-SOURCE
16	Johns et al. (2021)	“Can you hear me now?” Video conference coping strategies and experience during COVID-19 and beyond	Critical Sociology
17	Kushner (2021)	Eccentric gaze as a possible cause of “Zoom Fatigue”	Journal of Binocular Vision and Ocular Motility
18	Mamtani et al. (2021)	Impact of videoconferencing applications on mental health	BJPsych International
19	Massner (2021)	The use of videoconferencing in higher education	Communication Management, IntechOpen
20	Mutu et al. (2021)	The impact of the “Zoom Fatigue” phenomenon and ways of managing it	Lumen Proceedings: Vol. 15. 2nd International Conference Global Ethics—Key of Sustainability (GEKoS)
21	Nesher Shoshan und Wehrt (2021)	Understanding “Zoom fatigue”: A mixed-method approach	Applied Psychology
22	Ngien und Hogan (2022)	The relationship between Zoom use with the camera on and Zoom Fatigue: considering self-monitoring and social interaction anxiety	Information, Communication & Society
23	Nurismawan et al. (2022)	What is the counsellor’s role in overcoming the Zoom Fatigue phenomenon?	Proceedings of the International Seminar
24	Peper et al. (2021)	Avoid zoom fatigue, be present and learn	NeuroRegulation
25	Ratan et al. (2021)	Facial appearance dissatisfaction explains differences in Zoom Fatigue	Cyberpsychology, Behavior, and Social Networking
26	Riedl (2022)	On the stress potential of videoconferencing: definition and root causes of Zoom fatigue	Electronic Markets
27	Rößler et al. (2021)	Reducing videoconferencing fatigue through facial emotion recognition	Future Internet
28	Rump und Brandt (2020)	Zoom-Fatigue	Institut für Beschäftigung und Employability IBE
29	Salim et al. (2022)	Zoom fatigue and its risk factors in online learning during the COVID-19 pandemic	Medical Journal of Indonesia
30	Salsabila et al. (2021)	Zoom Fatigue on teacher and student’s communication pattern	Proceeding of the 1st International Conference on Social Sciences and Education
31	Shklarski et al. (2021)	Navigating changes in the physical and psychological spaces of psychotherapists during Covid-19: When home becomes the office	Practice Innovations
32	Shockley et al. (2021)	The fatiguing effects of camera use in virtual meetings: A within-person field experiment	Journal of Applied Psychology
33	Tobing et al. (2022)	Participants’ levels of understanding before and after attending the zoom fatigue during COVID-19 pandemic webinar	World Journal of Advanced Research and Reviews
34	Toney et al. (2021)	Fighting Zoom Fatigue: Keeping the zoombies at bay	Communications of the Association for Information Systems
35	Usta Kara und Ersoy (2022)	A new exhaustion emerged with COVID-19 and digitalization: A qualitative study on Zoom Fatigue	OPUS—Journal of Society Research
36	Wicks (2021)	Minimizing Zoom Fatigue and other strategies for a successful synchronous class experience	Tackling Online Education: Implications of Responses to COVID-19 in Higher Education Globally
37	Wiederhold (2020)	Connecting through technology during the coronavirus disease 2019 pandemic: Avoiding “Zoom Fatigue”	Cyberpsychology, Behavior, and Social Networking

## Footnotes

1 Im Gegensatz zu Riedl (2022) wird im vorliegenden Beitrag die Fachliteratur zu VCF-Bewältigungsstrategien systematisch nach Häufigkeiten der Maßnahmen klassifiziert. Zudem wurden in Riedl (2022) Bewältigungsstrategien exemplarisch (und somit nicht systematisch) klassifiziert, und zwar primär auf der Basis von Praxisberichten. Dies geschah deshalb, weil es zum Zeitpunkt des Verfassens des Beitrags von Riedl (2022), konkret Ende 2020 und Anfang 2021, noch kaum wissenschaftliche Fachliteratur gab, die auch Bewältigungsstrategien behandelte.
