# Supplementary material for: Videoconference Fatigue Coping Strategies
Source: HMD Prax Wirtsch Inform. 2023 Mar 30;60(6):1289–311. [Article in German] doi: 10.1365/s40702-023-00963-3 (PMC10062258; doi:10.1365/s40702-023-00963-3)
Supplement: Supplementary file 1 [file 40702_2023_963_MOESM1_ESM.pdf]

		Organisatorische Maßnahmen													
	Empirisch (E) Konzeptionell (K)	Pausen während und zwischen Meetings	Kamera deaktivieren/reine Audiokonferenzen	Interaktion durch Feedback, Reaktionen und Methodenwechsel	Beschränkung der Meetingdauer	Maßnahmen für Gesundheit und Wohlbefinden	ausreichendes technologisches Equipment und Infrastruktur	Reduktion der Meetinganzahl	Moderation, Organisation und Management der Meetings	Alternative Kommunikationsmittel	Verbesserung der VC-Fähigkeiten	Zeitpunkt des Meetings	Gruppenregeln	ausreichend Zeit zur Vorbereitung	Beachtung der Teilnehmerzahl
Amponsah et al. (2021)	E				x				x						
Asgari et al. (2021)	E	x			x										
Bailenson (2021)	K		x				x			x					
Bayindir/Gökce (2022)	E	x			x		x				x				
Bennett et al. (2021)	E	x	x									x	x		
Brown (2022)	K	x	x							x					
Brown Epstein (2020)	K	x	x	x			x	x	x	x	x				
Bullock et al. (2021)	K	x				x	x				x				
Chawla (2021)	E	x													
Collins (2020)	K		x												
(2022)	E			x											
Döring et al. (2022)	K	x		x	x	x	x		x		x		x		
Ebner/Greenberg (2020)	K	x	x	x											
Hall (2020)	K			x		x									
Hopf/Berger (2022)	E	x	x		x	x									
Johns et al. (2021)															
Kushner (2021)															
Mamtani et al. (2021)	K	x	x					x				x		x	
Massner (2021)	E	x		x				x							
Mutu et al. (2021)	K	x	x					x		x					
Nesher Shoshan/Wehrt (2021)	E						x		x						
Ngien/Hogan (2022)	E	x			x	x									
Nurismawan et al. (2022)															
Peper et al. (2021)	E			x											
Ratan et al. (2021)	E														
Riedl (2022)	K	x	x	x	x		x	x		x	x			x	
Rößler et al. (2021)	E			x					x						
Rump/Brandt (2020)	E	x			x				x						
Salim et al. (2022)	E	x			x	x									
Salsabila et al. (2021)	E			x											
Shklarski et al. (2021)	E	x				x						x			
Shockley et al. (2021)	E		x												
Tobing et al. (2022)	E					x									
Toney et al. (2021)	E			x											x
Usta Kara/Ersoy (2022)	E	x			x	x	x				x				
Wicks (2021)	K	x						x		x					
Wiederhold (2020)	K	x	x					x							
**Summe**		21	12	11	10	9	8	7	6	6	6	3	2	2	1

		Persönliche Maßnahmen											
	Empirisch (E) Konzeptionell (K)	Vermeidung von Multitasking	Bewegung	Blick direkt in die Kamera	Vermeidung von Ablenkungen und Unterbrechungen	Adaptierung des Arbeitsplatzes	Deaktivierung der Selbstansicht	Blick vom Monitor abwenden	Psychologische und soziale Unterstützung	Verwendung der Stummschaltung	frühzeitiger Login	Trennung beruflicher und privater Themen	Verbesserung der zwischenmenschlichen Beziehungen und Gruppenzugehörigkeit	Notizen mit Papier und Stift
Amponsah et al. (2021)	E				x									
Asgari et al. (2021)														
Bailenson (2021)	K						x							
Bayindir/Gökce (2022)	E		x											
Bennett et al. (2021)	E		x				x			x			x	
Brown (2022)	K	x			x		x							
Brown Epstein (2020)	K	x	x	x	x	x				x	x			
Bullock et al. (2021)	K	x			x	x		x	x			x		
Chawla (2021)														
Collins (2020)														
(2022)														
Döring et al. (2022)	K	x										x	x	
Ebner/Greenberg (2020)	K							x						
Hall (2020)	K							x						x
Hopf/Berger (2022)	E		x											
Johns et al. (2021)	E	x												
Kushner (2021)	E			x										
Mamtani et al. (2021)	K	x		x			x							
Massner (2021)	E				x									
Mutu et al. (2021)	K	x					x							
Nesher Shoshan/Wehrt (2021)	E													
Ngien/Hogan (2022)	E	x							x					
Nurismawan et al. (2022)	K								x					
Peper et al. (2021)	E		x	x	x	x		x						
Ratan et al. (2021)	E						x							
Riedl (2022)	K	x	x			x				x	x			
Rößler et al. (2021)														
Rump/Brandt (2020)	E													
Salim et al. (2022)														
Salsabila et al. (2021)	E		x											
Shklarski et al. (2021)	E		x						x					
Shockley et al. (2021)														
Tobing et al. (2022)														
Toney et al. (2021)														
Usta Kara/Ersoy (2022)	E	x	x	x		x		x	x					
Wicks (2021)	K	x		x	x									
Wiederhold (2020)	K	x		x		x				x				
**Summe**		12	9	7	7	6	6	5	5	4	2	2	2	1

		Technologische Maßnahmen			
	Empirisch (E) Konzeptionell (K)	Avatar, VR, AR und Telepräsenzroboter	Benutzung des "together mode"	Verwendung von Tools, die die Blickrichtung der Teilnehmer:innen korrigieren	Verkleinerung des Desktopfensters
Amponsah et al. (2021)	E				
Asgari et al. (2021)	E				
Bailenson (2021)	K				
Bayindir/Gökce (2022)	E				
Bennett et al. (2021)	E				
Brown (2022)	K				
Brown Epstein (2020)	K	x	x		x
Bullock et al. (2021)	K				
Chawla (2021)	E				
Collins (2020)	K				
de Oliveira Kubrusly Sobral et al. (2022)	E				
Döring et al. (2022)	K				
Ebner/Greenberg (2020)	K				
Hall (2020)	K				
Hopf/Berger (2022)	E				
Johns et al. (2021)					
Kushner (2021)					
Mamtani et al. (2021)	K				
Massner (2021)	E				
Mutu et al. (2021)	K				
Nesher Shoshan/Wehrt (2021)	E				
Ngien/Hogan (2022)	E				
Nurismawan et al. (2022)					
Peper et al. (2021)	E				
Ratan et al. (2021)	E	x			
Riedl (2022)	K	x	x	x	
Rößler et al. (2021)	E				
Rump/Brandt (2020)	E		x	x	
Salim et al. (2022)	E				
Salsabila et al. (2021)	E				
Shklarski et al. (2021)	E				
Shockley et al. (2021)	E				
Tobing et al. (2022)	E				
Toney et al. (2021)	E				
Usta Kara/Ersoy (2022)	E				
Wicks (2021)	K	x			
Wiederhold (2020)	K	x			
**Summe**		5	3	2	1
